# 3D-Volumetric Shunt Measurement for Detection of High-Risk Esophageal Varices in Liver Cirrhosis

**DOI:** 10.3390/jcm13092678

**Published:** 2024-05-02

**Authors:** Kathleen Glückert, Alexandra Decker, Jörn Arne Meier, Sebastian Nowak, Feras Sanoubara, Juliana Gödiker, Sara Noemi Reinartz Groba, Markus Kimmann, Julian A. Luetkens, Johannes Chang, Alois M. Sprinkart, Michael Praktiknjo

**Affiliations:** 1 Department of Internal Medicine I, University Hospital Bonn, 53127 Bonn, Germany; 2 Department of Internal Medicine B, University Hospital Münster, 48149 Münster, Germany; 3 Department of Diagnostic and Interventional Radiology, University Hospital Bonn, 53127 Bonn, Germany

**Keywords:** cirrhosis, varices, computed tomography, portal hypertension, volumetry

## Abstract

**Background and Objectives:** Esophageal varices (EV) and variceal hemorrhages are major causes of mortality in liver cirrhosis patients. Detecting EVs early is crucial for effective management. Computed tomography (CT) scans, commonly performed for various liver-related indications, provide an opportunity for non-invasive EV assessment. However, previous CT studies focused on variceal diameter, neglecting the three-dimensional (3D) nature of varices and shunt vessels. This study aims to evaluate the potential of 3D volumetric shunt-vessel measurements from routine CT scans for detecting high-risk esophageal varices in portal hypertension. **Methods:** 3D volumetric measurements of esophageal varices were conducted using routine CT scans and compared to endoscopic variceal grading. Receiver operating characteristic (ROC) analyses were performed to determine the optimal cutoff value for identifying high-risk varices based on shunt volume. The study included 142 patients who underwent both esophagogastroduodenoscopy (EGD) and contrast-enhanced CT within six months. **Results:** The study established a cutoff value for identifying high-risk varices. The CT measurements exhibited a significant correlation with endoscopic EV grading (correlation coefficient r = 0.417, *p* < 0.001). A CT cutoff value of 2060 mm^3^ for variceal volume showed a sensitivity of 72.1% and a specificity of 65.5% for detecting high-risk varices during endoscopy. **Conclusions:** This study demonstrates the feasibility of opportunistically measuring variceal volumes from routine CT scans. CT volumetry for assessing EVs may have prognostic value, especially in cirrhosis patients who are not suitable candidates for endoscopy.

## 1. Introduction

Advanced chronic liver disease (cALD) is a chronic disease that, regardless of its etiology, leads to a decreased life expectancy due to life-threatening complications and impaired liver function as the disease progresses [1]. Acute variceal bleeding due to the rupture of one of those varices represents a highly life-threatening complication in individuals with liver cirrhosis and cALD. Variceal bleeding not only leads to substantial morbidity and mortality but also presents a major challenge in the management of patients with advanced chronic liver disease. Regular monitoring is crucial for identifying the optimal time to initiate appropriate prophylaxis in order to prevent variceal bleeding or its recurrence. Over the natural progression of the disease, the portal venous pressure increases, which, in turn, leads to the development of esophageal or gastral varices [2]. Compensated advanced chronic liver disease (cACLD) [3] carries an annual risk of 7–8% for the development of gastroesophageal varices, and once small varices appear, the risk of progression to large varices is as high as 10–12% per year [4].

Routine visual endoscopic inspection and therapy using methods such as band ligation are critical components of variceal bleeding prophylaxis and preventing its recurrence. Currently, protocol upper gastrointestinal (GI) endoscopies remain the gold standard for evaluation and treatment of varices [5,6]. However, performing a routine esophagogastroduodenoscopy at set time intervals is invasive and requires sedation in some cases, which may cause potential complications in certain patients with cardiopulmonary pre-existing conditions [7]. Despite its significance, endoscopy is being performed less frequently according to the Baveno VI and Baveno VII guidelines in certain subset patients [8,9]. This reduction in endoscopies underscores the need for alternative non-invasive methods to monitor varices’ grades and extent due to the known nature of EV to progress over time.

In recent years, there has been a growing emphasis on the use of elastography as a diagnostic tool for liver diseases, specifically in the diagnosis of clinically significant portal hypertension (CSPH). Elastography is a non-invasive imaging technique that allows for the assessment of liver stiffness, which can be an indicator of liver fibrosis and, subsequently, portal hypertension. This method has gained significant attention due to its potential to provide valuable information about the severity and progression of liver diseases without the need for invasive procedures such as liver biopsies. A cutoff value of >15 kPa has been shown to be highly suggestive of cACLD [9].

With the Baveno VI and VII algorithms, the presence of high-risk varices can be ruled out for some patients based on elastography and laboratory cutoffs. However, a large grey zone with uncertain variceal status still remains [8,9]. Elastography is an important diagnostic tool almost exclusively used in specialized gastroenterology and hepatology care centers. Access to primary and secondary care providers with expertise in elastography remains limited, creating challenges for patients seeking timely and comprehensive care for gastrointestinal and hepatic conditions. This, again, leaves an urgent clinical need for readily available and accessible alternatives or complementary non-invasive methods to diagnose high-risk varices.

Computed tomography (CT) scans are routinely used in clinical practice in patients with liver cirrhosis. These scans are often performed because patients with cirrhosis require either surveillance or diagnostics scans. One of the major complications of cirrhosis is the development of hepatocellular carcinoma (HCC). According to the current guidelines, screening for HCC every 6 months via ultrasound with or without alfa-fetoprotein measurements is recommended. However, either in the case of patients with anatomical limitations for ultrasound such as obesity, or in the case of detection of a suspicious lesion with unclear dignity in ultrasound, another contrast-enhanced imaging modality such as computed tomography is indicated [10,11]. Aside from the indication mentioned above, CT scans are routinely performed during episodes of acute decompensation of patients suffering from liver cirrhosis as a tool for differential diagnosis.

In selected patients with recurrent or refractory ascites or variceal hemorrhage, the placement of a transjugular intrahepatic portosystemic shunt (TIPS) can improve survival [9]. In many gastroenterology departments, CT scans are performed routinely prior to TIPS placement to rule out thrombosis of the portal vein or HCC suspicious lesions. Similarly, many centers routinely indicate CT scans in the setting of evaluation for listing for liver transplantation in order to rule out contraindications. Furthermore, these scans provide valuable information about possible anatomical abnormalities to the surgeons performing the liver transplantation. Incidental findings during CT scans are common, and this opportunistic information can contribute to optimizing patient care by drawing more information of interest from these examinations [12,13].

Gastroesophageal varices and their extent can be visualized in contrast-enhanced CT scans in a non-invasive manner. Previous studies have primarily focused on measuring variceal diameter in CT scans to assess esophageal varices and, therefore, the corresponding risk of bleeding from these varices [14,15,16,17,18,19]. However, this one-dimensional measurement may not accurately represent the three-dimensional nature of varices and shunt vessels due to the changing diameter over the length of the vessel. In this study, we aim to evaluate the use of 3D volumetric measurements from routine CT scans as a more comprehensive method for detecting high-risk esophageal varices in patients with portal hypertension and known esophageal varices.

## 2. Materials and Methods

### 2.1. Patients and Data Collection

We included 142 patients from the University Hospital Bonn in this monocentric retrospective single-center study. The inclusion criteria were a diagnosed liver cirrhosis as well as a documented esophagogastroduodenoscopy (EGD) and CT scan in the hospital’s electronic medical records. All patients had both esophagogastroduodenoscopy (EGD) and contrast-enhanced CT scans performed within a six-month timeframe. The study period spanned from December 2015 to April 2016. Ethical approval was obtained from the institutional ethics committee with a waiver of written informed consent due to the retrospective nature of the study.

### 2.2. Endoscopy

EGDs were performed on patients with suspected or known portal hypertension. It was carried out by an internal medicine specialist with over six years of experience. The endoscopic assessment included grading the severity of esophageal varices based on the Paquet classification and the identification of macroscopic red spots as a correlate for an increased susceptibility for bleeding [20]. Variceal grade 0 indicated the absence of macroscopic varices. Patients were categorized into two groups: those with low-risk varices (EV grade 0–I) and those with high-risk varices (EV grade II–IV) based on the EGD results [20].

### 2.3. Imaging

**Figure 1:**
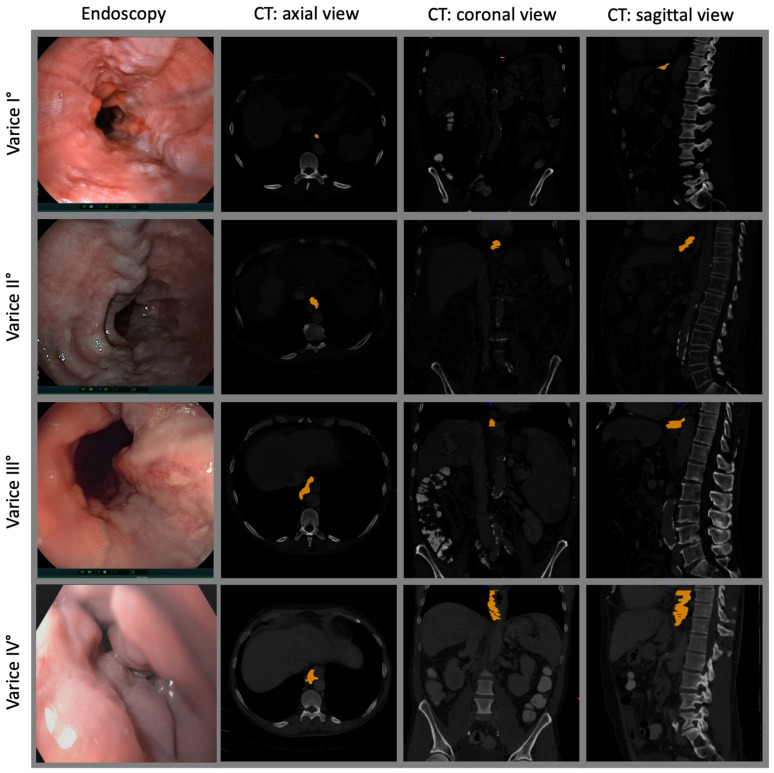
Examples of endoscopic and CT volumetry for variceal measurement.

Routine multislice CT scans with an iodinated contrast agent were performed on all patients within a six-month timeframe of EGD. Various models of Philips CT scanners in use by the radiology department of the University Hospital Bonn during the study period were used. Volumetric calculations were conducted using an in-house tool developed in MATLAB v9.6. The tool reconstructed axial, sagittal, and coronal images. Landmarks along the course of esophageal varices were manually determined. Volume was calculated by summing corresponding voxels and multiplying by voxel volume (Figure 1). To assess interrater reliability and retest reliability, a second measurement of a randomly selected subset of ten CT scans was performed at a time interval of over one year.

### 2.4. Statistical Analysis

The interrater reliability of the measured data points was evaluated using Pearson’s correlation coefficient. Continuous data is presented as the median (1st quartile–3rd quartile). Depending on the data distribution, nonparametric Mann–Whitney tests or parametric Student’s *t*-tests were used for group comparisons of continuous parameters. The correlation between CT volumetric analysis and endoscopic EV grading was evaluated using a chi-square test and binary logistic regression with ROC curve analysis. Based on the ROC curve, the AUC was calculated to evaluate the significance of the parameters in question. Also, based on the ROC curve, Youden’s Index was calculated to determine the cutoff value with the maximum sensitivity and specificity. A two-tailed *p*-value less than 0.05 was considered statistically significant.

## 3. Results

### 3.1. Study Population

The study included a total of 142 patients with liver cirrhosis; 56.3% of the entire study cohort was male, with both sexes being equally distributed in the low bleeding risk group. The high-risk bleeding group consisted of predominantly men (65.6%). The median age of all patients was 58 years, with a similar age distribution between subgroups. The two most common etiologies of liver cirrhosis were attributed to alcohol misuse (58%) and viral hepatitis (21%) in the overall cohort as well as in the subgroups. Of all the patients, 70.4% suffered from ascites, and 29.6% of all patients suffered from hepatic encephalopathy. Similar distributions could be observed between the low-risk and high-risk bleeding groups. The median MELD score of the entire study cohort was 13. The median in the low-risk group was 14 and was 11 in the high-risk group. The Child–Pugh score was similar, with a median of 7 in the overall cohort and both subgroups. The CLIF-C-AD score showed a similar distribution pattern, with 21 and 20 being the median of the entire study population as well as the subgroups.

Sodium and creatine distribution were similar between the study groups, while bilirubin showed a trend towards lower serum levels in patients within the high-risk bleeding subgroup (2.21 (0.21–18.78) vs. 1.51 (0.27–11.98), *p* = 0.075) without reaching statistical significance. The overall gGT was a median of 103. A statistically significant difference could be observed between the low-risk bleeding group and the high-risk group (90 (18–714) vs. 139 (22–585), *p* = 0.005). Patients within the high-risk group had higher serum gGT values. The other aminotransferases (AST and ALT) did not differ significantly between the study groups. Similarly, the liver synthesis parameters (Albumin and INR) did not differ between the subgroups. The blood-count parameters (WBC/Hb/Platelets) did not show a statistical difference between the low-risk and high-risk groups. As a bleeding prophylaxis, 53.5% of all patients were taking beta-blocker medication, and 16.9% had undergone TIPS placement. Both preventative measures were received by 12.7% of all patients. The distribution of those measures did not show a statistically significant difference between the study groups. Detailed patient characteristics are presented in Table 1.

### 3.2. Endoscopy

EGD identified esophageal varices in 103 patients (72.5%). Among them, 42 patients (29.6%) had grade I varices, and 48 patients (33.8%) had grade II varices. Eleven patients had grade III EV (7.7%) and two patients had EV grade IV (1.4%). Grade III and IV varices were less common in the study cohort. The patients were categorized into two groups based on their variceal grading: those with low-risk varices (EV grade 0–I, *n* = 81) and those with high-risk varices (EV grade II–IV, *n* = 61). Overall, general patient characteristics did not significantly differ between the two groups. Macroscopic red color signs were more common in the high-risk varices group (Table 1).

### 3.3. 3D Volumetry

Volumetric measurements were performed independently by two experts using the in-house MATLAB software tool as described previously. The measured shunt volume of the entire study cohort ranged from 82 mm^3^ to 55,793 mm^3^. Interrater reliability was excellent (r = 0.982). Shunt volume significantly increased with the higher grades of varices observed during endoscopy (*p* < 0.001, r = 0.417). Consequently, shunt volume was significantly larger in the high-risk varices group compared to the low-risk group (1419 (82–12,806) vs. 2125 (82–55,793), *p* < 0.001) (Table 2).

Binary logistic regression and ROC analysis were performed using shunt volume to predict the presence of high-risk varices, resulting in an AUC of 0.7 (Supplementary Figure S1). A shunt volume cutoff of 2060 mm^3^ (Youden’s index) was calculated to be the best for differentiating between small and large varices, with a sensitivity of 72.1% and a specificity of 65.4% (Table 3 and Table 4).

When applying his calculated cutoff, 70 patients in total had a shunt volume of less than 2060 mm^3^, of whom 53 had low-risk varices observed during endoscopy. Among the 72 patients with a shunt volume greater than 2060 mm^3^, 44 had high-risk varices (Table 4).

## 4. Discussion

In our study, we evaluated the feasibility of volumetric measurements of esophageal shunts from routine and opportunistic CT scans in patients suffering from liver cirrhosis at risk of EV development. We found a significant association between 3D shunt volume and traditional visual endoscopic grading of EV.

Non-invasive tools for early detection and grading of EV are essential for identifying cirrhosis patients who require close monitoring, especially regarding the Baveno VII guidelines. Although EGD is proven and well established, it is invasive and requires sedation which bears the risk of complications, especially sedation-related complications in patients with pre-existing cardiopulmonary conditions. EGD is still necessary for endoscopic therapy of varices, such as band ligation, but alternatives are needed for purely diagnostic purposes. Previous studies have demonstrated the benefit of CT in estimating high-risk EVs. Intriguingly, a recent meta-analysis showed that CT outperforms magnetic resonance imaging (MRI) and established LSM [21]. Most previous studies have investigated the role of variceal diameter, with diameters between three and five millimeters described as a critical threshold for high-risk varices. However, similar studies reported different cutoffs and a clear threshold to differentiate between low-risk and high-risk EVs has not yet been established [14,17,18]. For this reason, we hypothesized in this study that volumetric measurements may be a better marker for identifying varices, as they might be a better surrogate for a more accurate approximation of the blood pressure and blood flow within EVs. Our data demonstrates the effectiveness and reliability of 3D volumetric classification of EVs using CT measurements. A sensitivity of 72.1% and a specificity of 65.4% were achieved with a cutoff value (Youden’s index) of 2060 mm^3^. Wan et al. recently reported comparable values in a cohort of 136 patients, with an AUC of 0.768 (sensitivity 74.3%, specificity 71.4%) [22]. Future studies should evaluate 3D volumetric repeated measurements as guidance for therapy such as band ligation or the initiation of prophylaxis, such as NSBB. Utilizing solely the diameter of an EV as a seed visually in EGD disregards the three-dimensional nature of varices, as the diameter can vary greatly over the lengths of varices. One possibility to overcome this shortcoming is endoscopic ultrasound (EUS), which may complement the macroscopic aspect of EGD, as it allows for three-dimensional assessments [23,24]. However, due to the high level of expertise required to perform EUS proficiently, EUS is not widely available but is in specialized endoscopic centers. Therefore, CT or MRI scans may be more suitable alternatives, as both modalities are readily available to most patients.

CT using iodinated contrast agents is cost-effective and rarely associated with immediate complications, such as allergic reactions or extravasations. Furthermore, the radiation doses required have drastically been reduced with recent advances in CT imaging technology. Additionally, CT is already frequently used in patients with liver cirrhosis for several indications. This includes screening, confirmation, or surveillance of hepatocellular carcinoma as well as evaluation for TIPS placement. In patients eligible for liver transplantation, CT scans are routinely performed as part of the evaluation process. Furthermore, CT is routinely performed during episodes of acute decompensation of patients suffering from liver cirrhosis to assist in differential diagnosis and to assess liver status. Due to this multitude of reasons, opportunistic CT scans of liver cirrhosis patients are readily available and can provide additional information for risk stratification if analyzed as our study demonstrates.

Because the CT scan itself is not an examiner-dependent dynamic examination but rather an examiner-independent static examination, it allows for more observer-independent mapping of disease progression as opposed to complementary examinations such as EUS. One approach to making 3D volumetric shunt measurements independent of the examiner and the measurement method is the automatic evaluation of shunt volumes using specifically programmed software, as has been suggested [25]. This would allow for shunt measurement with each and every CT or MRI scan done, negating the need for invasive endoscopic intervention such as EGD or EUS. Moreover, it would lower the burden of work for radiologists and gastroenterologists who would have to perform the measurement manually, consuming valuable time that could otherwise be spent on patient care. Apart from this, manually marking EVs in axial and coronal CT images requires a high level of expertise, which makes an automatic, software-assisted process even more intriguing.

Our study has some limitations, including being a single-center retrospective study, which by design lends itself towards selection bias. Furthermore, we did not include a validation cohort. In addition, the dataset did not include clinical long-term outcomes, which could be interesting, especially in terms of shunt-associated hepatic encephalopathy [26]. Our sample size is also likely undersized and does not account for different etiologies of cirrhosis. Additionally, CT scans using iodinated contrast agents could potentially worsen kidney function in liver cirrhosis patients who tend to have impaired kidney function. This could potentially limit the application of this measurement. Moreover, our data do not include longitudinal measurements of 3D volumetry of varices, which was beyond the scope of this study.

However, the measurement’s reliability is high, with excellent inter-observer and intra-observer agreement for detecting EVs [27]. Moreover, it has been shown that radiologists and non-radiologists achieve comparable results, especially in advanced varices [28]. Further research using a large study cohort, preferably a multicenter study, is needed to define an optimal threshold for high-risk varices. Additionally, the predictive value of CT measurements for variceal bleeding remains unclear and should be evaluated in future studies [22,29,30]. Our data could not be used for this purpose because it was a retrospective study, and most of our patients received prophylactic treatment for variceal bleeding, which altered the natural progression of the disease.

## 5. Conclusions

This study demonstrates the feasibility of opportunistic measurement of variceal volumes from routine CT scans. CT volumetry for assessing EVs may have prognostic value, especially in cirrhosis patients who are not suitable candidates for endoscopy, particularly in centers that do not have VCTE available or access. VCTE should still be the first non-invasive method option, per the Baveno guidelines, since it has been validated in many studies. The 3D volumetric shunt CT still needs further validation in future studies.

## Figures and Tables

**Table 1:** Baseline characteristics.

Parameter		Baseline			
		All *n* = 142	Low Bleeding Risk *n* = 81	High Bleeding Risk *n* = 61	*p*-Value
Clinical	Age [years]	58 (35–79)	58 (35–79)	58 (37–79)	0.529
	Gender (male/female)	80/62 (56.3%/43.7%)	40/41 (49.4%/50.6%)	40/21 (65.6%/34.4%)	0.062
	Etiology of cirrhosis (alcoholic/viral/other)	83/30/29 (58.5%/21.1%/20.4%)	49/17/15 (60.5%/21.0%/18.5%)	34/13/14 (55.7%/21.3%/23.3%)	0.790
	Esophageal varices (0/I°/II°/III°/IV°)	39/42/48/11/2 (27.5%/29.6%/33.8%/7.7%/1.4%)	39/42/0/0/0 (48.1%/51.9%/0%/0%/0%)	0/0/48/11/2 (0%/0%/78.7%/18.0%/3.3%)	<0.001
	Red color signs	21 (14.8%)	3 (3.7%)	18 (29.5%)	<0.001
	Volume of shunt in CT [mm^3^]	2125 (82–55,793)	1419 (82–12,806)	3167 (284–55,794)	<0.001
	Ascites	100 (70.4%)	54 (66.7%)	46 (75.4%)	0.272
	Hepatic encephalopathy	42 (29.6%)	22 (27.2%)	20 (32.8%)	0.578
Scores	MELD	13 (5–38)	14 (5–38)	11 (6–33)	0.065
	MELD-Na	15 (5–38)	16 (7–38)	14 (5–35)	0.094
	Child–Pugh	7 (5–13)	7 (5–10)	7 (5–13)	0.105
	Child–Pugh grade [A/B/C]	49/80/13 (34.5%/56.3%/9.2%)	22/50/9 (27.2%/61.7%/11.1%)	27/30/4 (44.3%/49.2%/6.6%)	0.095
	CLIF-C-AD	21 (13–29)	21 (13–29)	20 (14–26)	0.634
Laboratory	Sodium [mmol/L]	137 (123–147)	137 (126–147)	137 (123–145)	0.591
	Creatinine [mg/dL]	0.98 (0.5–5.09)	0.93 (0.5–5.09)	1.0 (0.6–4.57)	0.172
	Bilirubin [mg/dL]	1.77 (0.21–18.78)	2.21 (0.21–18.78)	1.51 (0.27–11.98)	0.075
	gGT [U/L]	103 (18–714)	90 (18–714)	139 (22–585)	0.005
	AST [U/L]	47 (14–653)	46 (14–653)	54 (18–282)	0.441
	ALT [U/L]	30 (8–349)	29 (8–282)	31 (11–349)	0.421
	Albumin [g/L]	30 (18–50)	30 (18–50)	29 (18–46)	0.880
	INR	1.2 (0.9–3.4)	1.3 (0.9–3.4)	1.2 (0.9–3.0)	0.176
	WBC [G/µL]	5.7 (1.3–35)	5.7 (1.3–35)	5.5 (2.8–30.5)	0.918
	Hb [mg/dL]	10.5 (6.1–16.1)	10.8 (6.4–16.1)	9.9 (6.1–14.7)	0.195
	Platelets [G/µL]	113 (34–504)	119 (34–504)	109 (35–330)	0.543
Therapy	Prophylaxis (no/beta blocker/ ligation/both/ TIPS)	57/76/24/18/2 (40.1%/53.5%/16.9%/12.7%/1.4%)	34/41/10/7/1 (42.0%/50.6%/12.3%/8.6%/1.2%)	23/35/14/11/1 (37.7%/57.4%/23.0%/18.0%/1.6%)	0.569/0.394/0.103/0.103/0.841

CT, computed tomography.

**Table 2:** Correlation of volume of esophageal varices in CT [mm^3^] and endoscopic grading.

	EV 0° *n* = 39	EV I° *n* = 42	EV II° *n* = 48	EV III° *n* = 11	EV IV° *n* = 2	*p*	r
**Volume of shunt in CT [mm^3^]**	980 (82–12,806)	1870 (383–9647)	3000 (284–14,462)	7571 (1061–55,794)	9595 (985–18,203)	<0.001	0.417

CT, computed tomography; EV, esophageal varices; r—correlation coefficient.

**Table 3:** Sensitivity and specificity for different cutoffs of volume of shunt in CT [mm^3^].

Cutoff Volume of Shunt in CT [mm^3^]	Sensitivity	Specificity
2060	0.721	0.654
271	1	0.086
12,828	0.115	1

CT, computed tomography.

**Table 4:** Cross table of endoscopic and computed tomography classification of varices according to previously defined cutoffs.

	Endoscopic: Low-Risk Varices (EV 0°–I°)	Endoscopic: High-Risk Varices (EV II°–IV°)	
CT: small varices (<2060 mm^3^)	53	17	70
CT: large varices (>2060 mm^3^)	28	44	72
	81	61	142

CT, computed tomography; EV, esophageal varices.

## Data Availability

The data presented in this study are available on request from the corresponding author. The data are not publicly available due to GDPR.

## Supplementary Materials

The following supporting information can be downloaded at: https://www.mdpi.com/article/10.3390/jcm13092678/s1, Figure S1: ROC curve of the volume of shunt in CT [mm^3^] for discrimination of endoscopic small or large varices.

## Abbreviations

AUC	area under the curve
cACLD	compensated advanced chronic liver disease
cALD	Advanced chronic liver disease
CSPH	clinically significant portal hypertension
CT	computed tomography
EGD	esophagogastroduodenoscopy
EUS	endoscopic ultrasound
EV	esophageal varices
MRI	magnetic resonance imaging
LSM	liver stiffness measurement
ROC	receiver operating characteristic
TIPS	transjugular intrahepatic portosystemic shunt
NSBB	Non-selective beta blockers
