# Supplementary material for: 3D-Volumetric Shunt Measurement for Detection of High-Risk Esophageal Varices in Liver Cirrhosis
Source: J Clin Med. 2024 May 2;13(9):2678. doi: 10.3390/jcm13092678 (PMC11084829; doi:10.3390/jcm13092678)
Supplement: Supplementary file 1 [file jcm-13-02678-s001.zip › jcm-2945934-supplementary.pdf]

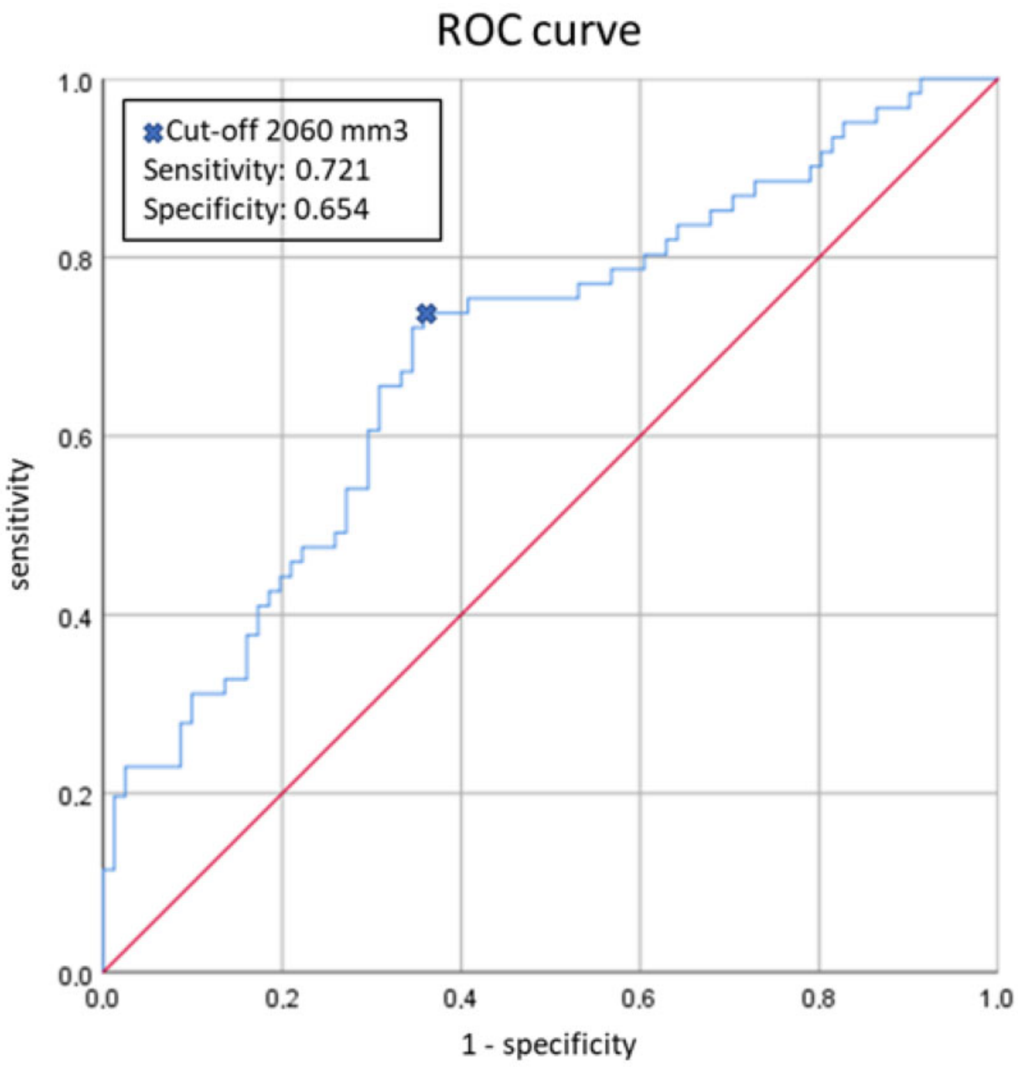

Supplementary Figure S1: Receiver operating characteristic (ROC) curve of volume of shunt in CT [mm^3] for the discrimination of endoscopic small or big varices.
